# Standard abdominal wound edge protection with surgical dressings vs coverage with a sterile circular polyethylene drape for prevention of surgical site infections (BaFO): study protocol for a randomized controlled trial

**DOI:** 10.1186/1745-6215-13-57

**Published:** 2012-05-15

**Authors:** André L Mihaljevic, Christoph W Michalski, Mert Erkan, Carolin Reiser-Erkan, Carsten Jäger, Tibor Schuster, Christoph Schuhmacher, Jörg Kleeff, Helmut Friess

**Affiliations:** 1 Department of Surgery, Klinikum Rechts der Isar, Technische Universität München, Ismaningerstrasse 22, Munich, 81675, Germany; 2 CHIR-Net. Munich, Munich, Germany; 3 Institute for Medical Statistics and Epidemiology, Klinikum Rechts der Isar, Technische Universtität München, Ismaninger Str. 22, Munich, 81675, Germany

**Keywords:** Abdominal dressing, Abdominal surgery, Randomized trial, Surgical site infection, Wound edge protector, Wound infection

## Abstract

**Background:**

Postoperative surgical site infections cause substantial morbidity, prolonged hospitalization, costs and even mortality and remain one of the most frequent surgical complications. Approximately 14% to 30% of all patients undergoing elective open abdominal surgery are affected and methods to reduce surgical site infection rates warrant further investigation and evaluation in randomized controlled trials.

**Methods/design:**

To investigate whether the application of a circular plastic wound protector reduces the rate of surgical site infections in general and visceral surgical patients that undergo midline or transverse laparotomy by 50%. BaFO is a randomized, controlled, patient-blinded and observer-blinded multicenter clinical trial with two parallel surgical groups. The primary outcome measure will be the rate of surgical site infections within 45 days postoperative assessed according to the definition of the Center for Disease Control. Statistical analysis of the primary endpoint will be based on the intention-to-treat population. The global level of significance is set at 5% (2 sided) and sample size (n = 258 per group) is determined to assure a power of 80% with a planned interim analysis for the primary endpoint after the inclusion of 340 patients.

**Discussion:**

The BaFO trial will explore if the rate of surgical site infections can be reduced by a single, simple, inexpensive intervention in patients undergoing open elective abdominal surgery. Its pragmatic design guarantees high external validity and clinical relevance.

**Trial registration:**

http://www.clinicaltrials.gov NCT01181206. Date of registration: 11 August 2010; date of first patient randomized: 8 September 2010

## Background

### Rationale for the trial

Postoperative surgical site infections (SSIs) are one of the most frequent surgical complications and a major cause of postoperative morbidity, prolongation of hospital stay, healthcare costs and even mortality. An estimated 300,000 to 500,000 SSIs occur in the USA annually [1-4]. In Germany SSIs are the third most frequent cause of nosocomial infections and account for approximately 15% of the 400,000 to 600,000 nosocomial infections per year (60,000 to 90,000 cases) according to data from the Robert-Koch Institute (RKI) and the German Nationales Referenzzentrum für Surveillance von Nosokomialen Infektionen (NRZ) [5,6]. Data from the German Krankenhaus Infektions-Surveillance-System (KISS) estimate an even higher number of approximately 128,000 SSIs annually [6]. Furthermore, an estimated 7,500 to 15,000 patients die every year in Germany due to nosocomial infections [7,8].

Despite the implementation of preventive measures such as preoperative antibiotic prophylaxis [9-12] and antiseptic skin cleansing [9,13], SSI rates in prospective trials with adequate follow-up and standardized SSI definition in abdominal surgical patients remain high and vary from 14% to 32% [14-17]. Applying the SSI criteria of the Center for Disease Control (CDC) [9], which differentiate between superficial, deep and organ-space SSIs at postoperative day 30, Darouiche *et al*. reported an overall SSI rate of 16.1% in the control group (povidone-iodine skin preparation) of their randomized controlled trial (RCT) in patients with clean-contaminated abdominal surgery [18]. Seiler et al. reported similar numbers in a randomized multicenter trial comparing three different techniques of abdominal wall closure in abdominal surgical patients with SSI rates varying from 13% to 19% [19]. SSI rates following colorectal surgery seem to be even higher as was shown by Bennett-Guerrero and colleagues, who reported an overall SSI rate of 21% in the control group (and 30% in the intervention group) of their prospective multicenter RCT comparing the application of a gentamicin-collagen sponge versus no intervention in colorectal patients [20]. National data from the USA support these numbers [21].

Multiple studies have shown an increase in the mean length of hospital stay by 6 to 24 days if SSIs occur [4,22-25]. The resulting direct costs have to be added to the indirect costs such as loss of workforce or insurance payments resulting in substantial expenses for the healthcare system [26-28].

The most frequent pathogens causing postoperative SSIs in general and abdominal surgical patients are endogenous pathogens from the skin or gastrointestinal tract (KISS data 2005 to 2008, [29]). This implies that adequate protection of the surgical site by wound edge protectors might reduce the rate of postoperative SSIs.

### Previous trials

Several previous trials have investigated the effect of wound edge protectors on SSI rates in abdominal surgery and report mixed results. While some found beneficial effects [30-33] others reported no benefit [34-36]. Similarly, a randomized trial in 2011 in which wound protectors were introduced together with other measures as a bundle to reduce SSIs did not show a benefit [37]. The diverging results may be explained by trial design, since the mentioned trials were either performed in a single institution setting, had small sample sizes, lacked adequate blinding, standardization or used varying definitions of outcome variables (SSI definitions). Large multicenter randomized trials comparing the use of a wound edge protector with standard intervention (sterile towels) under standardized conditions and with defined outcome variable (CDC definition of SSIs) are lacking.

**Figure 1:**
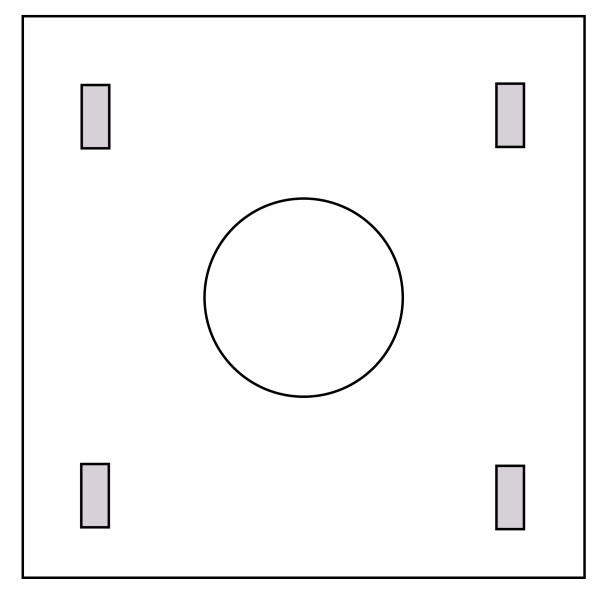
**Schematic drawing of the wound edge protectors used in the BaFO trial.** The protector consists of an impervious drape that covers the abdomen of the patient and is fixed to the skin with the help of adhesive tapes. It has a standardized size of 90 × 90 cm. The hole in the middle is connected to a flexible plastic ring that fits into the abdominal wound and protects the wound edges (skin, subcutaneous tissue, fascia and muscle) from contact with viscera, visceral contents and gloves, while allowing for visualization of the wound edges.

It is important to point out that BaFO will apply a circular plastic wound edge protector covering the whole width of the wound (skin, subcutaneous tissue, fascia and muscle; Figure 1) not other forms of surgical draping such as adhesive incise drapes for which beneficial [38,39] as well as non-beneficial results [40,41] have been reported. A Cochrane meta analysis of the latter intervention compared to no adhesive drapes found no evidence that iodine impregnated adhesive drapes reduce the SSI rate (relative risk 1.03, 95% confidence interval 0.064 to 1.66, *P* = 0.89) [42]. Interestingly, when the authors of the same meta analysis compared the use of non-impregnated adhesive drapes to no drape usage, they found the SSI rate significantly increased in the drape group (relative risk 1.23, 95% confidence interval 1.02 to 1.48, *P* = 0.03).

### Objective

BaFO aims to investigate whether the application of a circular plastic wound edge protector (Figure 1) reduces the rate of SSIs (within 45 days postoperatively) in abdominal surgical patients that undergo elective midline or transverse laparotomy by 50% (from 16% to 8%). As a secondary endpoint the difference in intraoperative core body temperature between the two study groups will be evaluated.

### Trial sites

The BaFO trial will be performed at 15 sites of the Trial Network (CHIR-*Net*) of the German Surgical Society (Deutsche Gesellschaft für Chirurgie (DGCH)). Most of these sites have participated in previous randomized controlled trials and all centers were adequately trained and prepared according to International Conference on Harmonisation of Technical Requirements for Registration of Pharmaceuticals for Human Use (ICH)-good clinical practice (GCP) rules for participation in this trial. CHIR-*Net* is funded by the German Ministry of Research and Education (Bundesministerium für Bildung und Forschung (BMBF); see funding information below).

## Methods/design

### Trial population and eligibility criteria

All general and abdominal surgical patients scheduled for elective open abdominal surgery requiring a median or transverse laparotomy will be eligible, given their ability to understand the extent and nature of the BaFO trial as well as their written informed consent. Patients participating in the BaFO trial must be 18 years of age or older, and the planned operation should be classified as clean or clean-contaminated as per the CDC definition [9]. Exclusion criteria are listed in Figure 1.

**Table 1:** Exclusion criteria of the BaFO trial

**No.**	**Exclusion criteria**
1	ASA grade >3
2	Pregnant or lactating women
3	Midline or transverse laparotomy within the last 60 days prior to trial intervention
4	Planned relaparotomy within 30 days after trial intervention
5	Contaminated operations according to CDC definition [9]
6	Small abdominal operations without planned transverse or midline laparotomy (for example, appendectomy)
7	Concurrent abdominal wall infections
8	Severe immunosuppression, for example after: organ or bone marrow transplantation, concurrent steroid treatment with >10 mg prednisone daily (or an equivalent dose of any other steroid), concurrent infliximab treatment or treatment with an equivalent immunosuppressive substance, or chemotherapy within the last 2 weeks prior to trial intervention
9	Severe preoperative neutropenia (≤0.5 × 10^9^ cells/l)
10	Liver cirrhosis; Child-Pugh B or C [43]

ASA = American Society of Anesthesiologists; CDC = Centers for Disease Control and Prevention.

### Sample size

A total of 258 patients will be analyzed per group. Given an estimated drop out rate of approximately 15%, 600 patients will be randomized to 1 of the 2 treatment arms.

### Type of trial

Randomized, controlled, observer and patient blinded multicenter surgical trial with two parallel comparison groups.

### Recruitment and trial timeline

A total of 15 centers of general and abdominal surgery in Germany will participate in this trial. The centers vary from university hospitals to community hospitals and include certified centers for colorectal surgery (Darmzentren). All centers are members of the Trial Network (CHIR-*Net*) of the German Surgical Society. Physicians or nurses involved in the trial have been trained in ICH-GCP prior to initiation of the trial. Furthermore, all centers and participants were specifically instructed in study-specific procedures prior to the start of the trial. The centers will be supported by an ICH-GCP qualified flying study nurse from the CHIR-*Net* Surgical Regional Centre Munich to ensure protocol conforming data acquisition and trial interventions. Stratification according to center will be performed and all centers must recruit a minimum of 15 patients.

**Figure 2:**
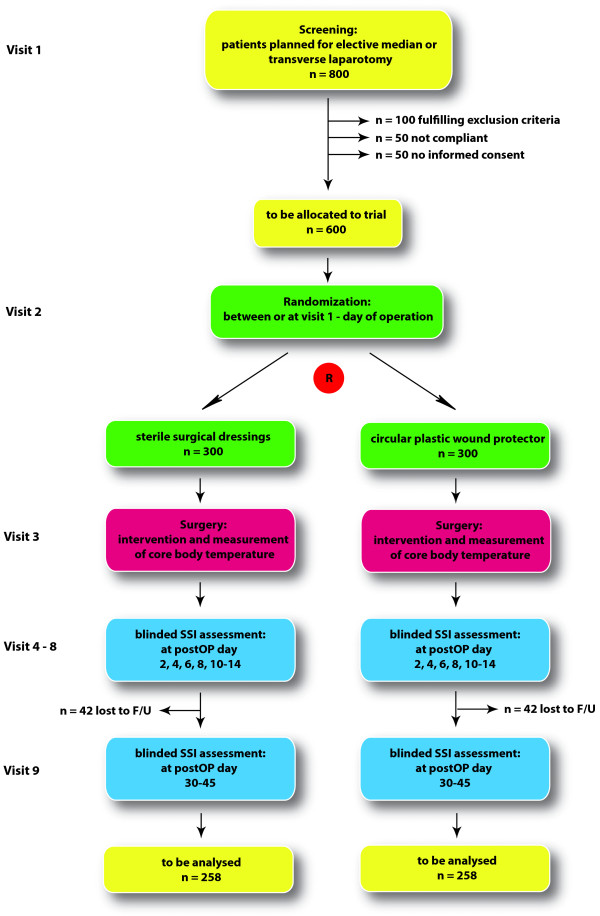
**Flow chart of the BaFO trial.** F/U = follow-up; postop day = postoperative day; R = randomization; SSI = surgical site infection.

The duration of the recruitment phase is expected to be 30 months. The last follow-up will be performed a maximum of 45 days after the last patient underwent the trial intervention. Hence, the total duration of the trial is expected to be no longer than 32 months. The study flow is outlined in Figure 2.

### Randomization and blinding

Randomization and blinding will be performed with the help of sealed, opaque, individually numbered envelopes, restricted to choosing one at a time. The envelopes contain data sheets with information regarding the group allocation and the randomization number. Both will be prefabricated by a biostatistician of the Technische Universität München (TUM; Munich, Germany). Randomization (visit 2, see Figure 2) will be performed after inclusion of the patient in the trial (informed consent, visit 1) or in the period between inclusion and the trial intervention (day of surgery) or at the day of surgery (visit 3). Randomization will be performed stratified by center. To assure balanced group sizes in the course of the accrual, a blockwise randomization is applied. Basic characteristics of the patient and day of randomization must be documented on the randomization sheets. Subsequently, randomization sheets must be dated, signed and stored away from the patient records, the trial documents and the investigator site file to ensure blinding. Patients, outcome assessors and the trial statistician will be blinded for the trial intervention. The outcome assessor (postoperative SSIs) will therefore neither be part of the surgical team that performs the trial intervention nor have access to the randomization sheets. Patients will be blinded since they are under general anesthesia during the operation and therefore will not be aware which intervention is used during the operation.

### Interventions

Experimental intervention: all elective general or abdominal surgical patients undergoing a midline or transverse laparotomy for any cause will have their surgical incisions covered with a circular sterile plastic wound edge protector during the operation (see Figure 1).

Control intervention: draping of the surgical incision will be performed with standard sterile surgical dressings.

The schedule of trial interventions is presented in Figure 2.

**Table 2:** Study visits of the BaFO trial

**Activity**	**Visit 1 (screening)**	**Visit 2 (randomization)**	**Visit 3 (operation)**	**Visit 4 (postop day 2)**	**Visit 5 (postop day 4)**	**Visit 6 (postop day 6)**	**Visit 7 (postop day 8)**	**Visit 8 (postop days 10 to 14)**	**Visit 9 (postop days 30 to 45)**
Demographics and baseline clinical data	X								
Inclusion/exclusion	X								
Physical examination	X								
Patient information and informed consent	X								
Blood sampling^a^	X								
Randomization		X							
Temperature measurement			X						
Wound documentation				X	X	X	X	X	X
Mibi swab^c^				X	X	X	X	X	X
Measurement of wound diameter				X					

^a^Blood sampling: Serum: sodium, potassium, creatinin, C-reactive protein, total protein, blood urea nitrogen, glutamic-pyruvic transaminase, glutamic-oxaloacetic transaminase, γ-glutamyl transpeptidase, pseudocholinesterase, alkaline phosphatase, glucose; blood count: leucocytes, erythrocytes, hemoglobin, hematocrit, mean corpuscular hemoglobin, mean corpuscular volume, mean corpuscular hemoglobin concentration, platelets; Coagulation: quick (international normalized ratio), prothrombin time.

^b^One blood sampling between postoperative day 4 to 8 (visits 5 to 7).

^c^In case of a surgical site infection, a microbiological swab according to local practice should be obtained for microbiological specification and antimicrobial testing.

### Risks

No additional risks for study patients are anticipated, since the application of a circular sterile plastic wound edge protector represents a clinically established standard method. The wound edge protector used in BaFO is CE certified (3 M Steri-Drape Wound Edge Protector, 3 M, St. Paul, MN, USA). All surgical procedures carried out within BaFO are not affected by the trial.

### Outcomes

Primary efficacy endpoint of the BaFO trial is the rate of SSIs according to Center for Disease Control (CDC) criteria, which constitutes the internationally accepted standard definition within 45 days postoperatively [9]. Secondary outcome measure is the intraoperative core body temperature measured at the beginning, in the middle and at the end of the operation via nasal, rectal, transurethral or central venous probes. Temperature as well as the modality of measurement will be recorded.

### Data management

All required information collected during the trial is entered in the case record form (CRF) by the investigator or a designated representative. Documentation is expected to be completed as soon as possible after the information has been collected. The investigator is responsible for the accuracy of the documentation and must ensure that all entries can be verified by source data. An explanation must be given for all missing data. Corrections in the CRF must be signed, dated and leave the corrected entry visible. The completed CRF must be reviewed, signed and dated by the investigator named in the trial protocol or by a designated subinvestigator. After keeping a copy at the trial center, the original CRF is sent in a sealed envelope by certified mail to the Centre for Data Management at the Münchner Studienzentrum (MSZ; member of the Network of Coordinating Centres for Clinical Trials (KKS Network) at the Technische Universität München, Munich, Germany). Double data entry is performed by data management according to standard operating procedures predefined in the data management plan to ensure correct transfer of data from the CRF to the database (hardware: Windows Server NT 2003 (Microsoft, Redmond, WA, USA); software: MACRO™ V.3, Microsoft SQL Database (Microsoft, Redmond, WA, USA); browser: Microsoft Internet Explorer V.6 or higher (Microsoft, Redmond, WA, USA)). Completeness, validity and plausibility of data are examined by validating programs as well as individual inspection and queries are generated accordingly which need to be clarified by the investigator or designated subinvestigator. At the end of the trial the principal investigator will retain the original CRFs.

### Monitoring

Monitoring of the trial data will be performed by an independent institution experienced in the monitoring of surgical trials (KKS Network) at the TUM (Münchner Studienzentrum). Monitoring will be carried out in accordance with ICH-GCP guidelines [44] and standard operating procedures of the MSZ to ensure patients´ safety and integrity of the clinical data, for example, primary outcome measure in adherence to study protocol. All trial sites are activated with an initiation visit by the monitor or CHIR-*Net* coordinator, who will hand out and explain the investigator site file, discuss relevant issues and train trial personnel in study-specific interventions. Regular contact by phone or email with all participating centers will enable the CHIR-*Net* Coordinator and the monitor to control the study progression, adherence to the study protocol, and to discuss problems related to the study. Regular on-site monitoring visits are planned for all sites. Investigators must allow the monitor to look at all essential documents, support the monitor during visits and answer queries. All monitoring procedures will be predefined in a trial-specific monitoring manual. In addition, a GCP-trained flying study nurse employed by the CHIR-*Net* regional center Munich will assist the trial sites with documentation and data collection if needed. Furthermore, close-out visits are planned for each center.

### Safety evaluation and reporting of adverse events

Occurrence of the primary endpoint is assessed as endpoint only (not as adverse event).

The following conditions are expected after the initial operation and will therefore not be classified as adverse events: pain, nausea, vomiting, urinary tract infection, hyper/hypotension, imbalances of blood sugar or electrolytes and other lab values out of range, if they do not exceed grade 3 to 4 in the Common Terminology Criteria for Adverse Events version 3.0 [45]. Assessment will be performed by the investigator or the designated subinvestigator.

From the day the patient has signed informed consent until the regular end of the trial (visit 9) or until premature withdrawal of the patient, all serious adverse events (SAEs) will be documented on a ‘serious adverse events form’ available in the investigator site file. An SAE will be defined as an event, that results in death, is immediately life threatening, requires or prolongs hospitalization or results in persistent or clinically important disability or incapacity as judged by the investigator or designated subinvestigator. SAEs will be classified to intensity (mild, moderate, severe), outcome (ongoing, recovered completely, recovered with sequelae, death, unknown) and causality (unrelated; possibly, probably or definitely related to trial intervention; not assessable). The assessment is based on clinical findings and needs to be performed by the investigator or designated subinvestigator in the participating trial centers. SAEs will have to be reported within 7 days after becoming known.

### Statistical methods

#### Sample size calculation

Sample size calculation is based on the primary end point of surgical site infections within 45 days post operation according to the CDC classification [9] and was conducted by using nQuery Advisor software version 7.0 (Statistical Solutions Ltd, Cork, Ireland). Based on the assumption that the percentage of patients developing postoperative wound infections in a mixed surgical population undergoing midline or transverse laparotomy is approximately 16% for the standard group (control group, see Background) and can be reduced to 8% in the experimental intervention arm, a group sample size of 258 patients would need to be compared by the χ^2^ test, to achieve 80% power in detecting this difference in SSI rate at a 2-sided level of significance of 5%. Analysis of the primary efficacy endpoint will be based on a χ^2^ and not based on a random effects model, which would assume different underlying effects within the centers. However a generalized linear mixed effect model (logit link regression) will be fitted to the data in terms of a supportive sensitivity analysis that allows for assessment of the primary efficacy endpoint under consideration of random center effects. Under the assumption of a dropout rate of up to 15%, a total of 600 patients would need to be enrolled in the study. Due to the broad inclusion criteria, the limited number of exclusion criteria, the limited time frame as well as the comprehensible nature of the trial, no more than 200 patients are expected to be screening failures, which brings the total number of patients that need to be screened for eligibility to 800 (Figure 2).

Based on the multicenter trail design, stratification according to recruiting center will be used [46]. Each participating center will have to enroll a minimum of 15 patients. Centers with fewer than 15 patients will be excluded from the primary efficacy analysis. Therefore, recruitment will be continued until enough centers lead to the required total sample size by each enrolling a sufficient number of patients.

#### Analysis

The primary analysis will be conducted according to the intention-to-treat (ITT) principle, that is, all included patients will be analyzed as randomized. Patients with missing primary outcome data will be considered as non-SSI cases if they belong to the control group and as SSI cases if they were randomized to the innovative treatment arm. For purpose of sensitivity analyses, per protocol and complete case analyses of the primary endpoint will be conducted and further appropriate and less conservative missing-value replacement strategies such as multiple conditional imputation [47] will be employed.

A planned interim analysis will be performed after recruitment and follow-up of 340 patients (two-thirds of the planned total). Due to this intended interim analysis, global α error adjustment is performed by the method of O’Brien and Fleming [48]. Therefore, evaluation of the primary efficacy endpoint will be conducted at a 0.005 level of significance (two sided) in the interim analysis and at a 0.048 level of significance (two sided) in the final statistical analysis.

Prespecified subgroup analyses or treatment group comparisons will be performed for: rate of superficial postoperative SSIs (according to CDC classification); rate of deep postoperative SSIs (according to CDC classification); rate of postoperative SSIs of organ space (according to CDC classification); rate of postoperative SSIs (total/superficial/deep/organ space) stratified by the National Nosocomial Infection Surveillance (NNIS) risk score [46]; rate of postoperative SSIs (total/superficial/deep/organ space) stratified by colorectal and non-colorectal operations.

Secondary analyses will be conducted in an explorative manner. Kaplan-Meier analysis and Cox regression analyses will be employed for analyses of time to event endpoints. The χ^2^ test will be used to compare frequency data between intervention groups. As appropriate, the Student t test, Mann–Whitney U test or analysis of covariance (ANCOVA) will be employed for group comparisons of quantitative data. The 95% confidence intervals will be provided for estimates of relevant effect sizes.

Safety analysis includes description and comparison of the frequency of adverse and serious adverse events in the two intervention groups.

Procedures for the statistical analysis of the primary and secondary endpoints will be conducted in line with the ICH-GCP E9 guideline [49]. For the statistical analysis, SAS® software version 9.2 or higher will be used (SAS Institute Inc., Cary, NC, USA). The statistical analysis will be performed by a group-allocation-blinded statistician from the Institute for Medical Statistics and Epidemiology of the TUM.

### Withdrawals

Patients are free to withdraw trial participation at their own request at any time and without giving reasons for their decision. Withdrawals will be documented in the CRF and in the patient´s medical record. Furthermore, all ongoing SAEs have to be followed-up and documented until their final outcome can be determined.

### Stopping guidelines

The trial can be prematurely closed by the coordinating investigator in consultation with the responsible biostatistician for the following reasons: the planned interim analysis indicates that rate or severity of SAE/morbidity in this trial poses a potential health hazard caused by the trial treatment in one or both of the trial groups; it appears that patients' enrolment is unsatisfactory with respect to quality or quantity or data recording is severely inaccurate or incomplete; external evidence demanding a termination of the trial.

In case of premature closure, the ethics committee has to be informed.

### Trial organization and administration

#### Funding

The Trial Network CHIR-*Net* of the German Surgical Society (DGCH) is funded by the German Ministry of Research and Education (Bundesministerium für Bildung und Forschung (BMBF); 01GH0702). The circular polyethylene wound protector (Steri-Drape Wound Edge Protector) used in the BaFO trial is provided by 3 M Infection Prevention Division (3 M Medica, 3 M Deutschland GmbH, Neuss, Germany). No financial support is given other than the funding by the BMBF mentioned above (01GH0702). There are no restrictions on publications and no conflict of interest. The idea for the BaFO trial was conceived, the trial protocol written and the trial initiated independent of the any industrial funder. Industrial funders and trial management are independent.

#### Ethics approval

Before the start of the trial, the trial protocol, informed consent document and any other trial documents were submitted to the independent ethics committee on 7 June 2010. Ethics approval was granted on 22 June 2010. A major protocol amendment was submitted to the ethics committee on 18 November 2010 and was approved 1 December 2010.

#### Registration

The trial protocol was registered at http://www.clinicaltrials.gov and was given the number NCT01181206.

#### Good clinical practice

The procedures set out in this trial protocol, pertaining to the conduct, evaluation and documentation of this trial, are designed to ensure that all persons involved in the trial abide by Good Clinical Practice [44] and the ethical principles described in the current revision of the Declaration of Helsinki [50]. The trial will be carried out in keeping with local legal and regulatory requirements.

## Discussion

Postoperative SSIs are among the most frequent surgical complications affecting approximately 14% to 32% [14-17] of abdominal surgical patients. These numbers have changed little over the last 20 years despite internationally accepted recommendations for control of SSIs (reviewed in [51]). In prospective trials with clear definitions, SSI rates tend to be higher [14,18-20] due to the fact that a number of SSIs occur late, after discharge of the patients from the hospital and would thus remain unnoticed if standardized wound evaluation as well as adequate follow-up are not applied. Furthermore, even if superficial SSIs are diagnosed, inadequate follow-up will result in underestimation of the number of deep or organ-space SSIs. To standardize reporting of SSI rates, the CDC has issued an internationally accepted definition of SSIs. According to this definition SSIs are grouped into superficial, deep and organ-space SSIs (Figure 3).

**Table 3:** **Definitions of abdominal surgical site infections (SSIs) classified according to the Centers for Disease Control and Prevention**[9]

**Classification**	**SSI type**
	Superficial incisional SSI:
1	Infection occurs within 30 days after the operation AND
2	Infection involves only skin or subcutaneous tissue of the incision AND
3	At least one of the following:
	A. Purulent drainage, with or without laboratory confirmation, from the superficial incision
	B. Organisms isolated from an aseptically obtained culture of fluid or tissue from the superficial incision
	C. At least one of the following signs or symptoms of infection: pain or tenderness, localized swelling, redness, or heat and superficial incision is deliberately opened by surgeon, unless incision is culture negative
	D. Diagnosis of superficial incisional SSI by the surgeon or attending physician
	Deep incisional SSI:
1	Infection occurs within 30 days after the operation AND
2	Infection involves deep soft tissues (for example, fascial and muscle layers) of the incision AND
3	At least one of the following:
	A. Purulent drainage from the deep incision but not from the organ/space component of the surgical site
	B. A deep incision spontaneously dehisces or is deliberately opened by a surgeon when the patient has at least one of the following signs or symptoms: fever (>38°C), localized pain, or tenderness, unless site is culture negative
	C. An abscess or other evidence of infection involving the deep incision is found on direct examination, during reoperation, or by histopathologic or radiologic examination
	D. Diagnosis of a deep incisional SSI by a surgeon or attending physician
	Notes: (1) report infection that involves both superficial and deep incision sites as deep incisional SSI; (2) report an organ/space SSI that drains through the incision as a deep incisional SSI
	Organ/space SSI:
1	Infection occurs within 30 after the operation AND
2	Infection involves any part of the anatomy (for example, organs or spaces), other than the incision, which was opened or manipulated during an operation AND
3	At least one of the following:
	A. Purulent drainage from a drain that is placed through a stab wound^a^ into the organ/space
	B. Organisms isolated from an aseptically obtained culture of fluid or tissue in the organ/space
	C. An abscess or other evidence of infection involving the organ/space that is found on direct examination, during reoperation, or by histopathologic or radiologic examination
	D. Diagnosis of an organ/space SSI by a surgeon or attending physician

SSIs are categorized into superficial, deep and organ-space infections.

^a^If the area around a stab wound becomes infected, it is not an SSI. It is considered a skin or soft tissue infection, depending on its depth.

The most frequent pathogens causing postoperative SSIs in general surgical patients are *Staphylococcus aureusEscherichia coli* and *Enterococcus* spp. Similarly, in abdominal surgical patients *E. coliEnterococcus* spp, *Enterobacter* spp and *S. aureus* are the most frequent pathogens (KISS data 2005 to 2008, [29]). These data indicate that endogenous infections from the patients´ skin or the gastrointestinal tract account for most SSIs and that a high number of SSIs might be prevented by adequate coverage of the incisional wound edges during surgical procedures. Wound edge protectors such as the one used in the BaFO trial do not only prevent displacement of skin pathogens into the surgical site such as incisional drapes do, but also effectively protect the skin, subcutaneous tissue, fascia and muscle from spillage of abdominal content during the surgical procedure. In addition, wound edge protectors cover the entire abdomen during the surgical procedure and thus have the theoretical benefit of improved temperature control and prevention of intraoperative hypothermia, a factor associated with SSIs in several studies [52,53]. We have incorporated this question into the BaFO trial by measuring the intraoperative core body temperature in both groups and analyze the differences as secondary endpoint.

However, wound edge protectors have not yet been rigorously tested in multicenter randomized controlled trials, since the studies available today are either single-center trials, lack clear SSI definitions/endpoints, or only include a small number of patients. Even in the trials available mixed results have been reported. A recent single center trial in elective colorectal patients reported a reduction of SSI rates from 22.7% to 4.7% [30]. Similarly, in a non-randomized single-center prospective trial with 221 patients reported a reduction in SSI rates only for patients undergoing colorectal surgery. Interesting only superficial SSI rates were reduced while rates of organ-space infections remained unchanged [31]. A randomized trial with 352 patients at 2 institutions found a reduction of SSIs from 22.6% to 10.5% with the application of a wound-edge protector in patients with clean/contaminated or contaminated wounds [32]. However, in a report from Psaila *et al*. wound-edge protection did not reduce SSI rate in abdominal surgery [34]. Similarly, a study by Kercher *et al*. reported no benefit of wound protectors in patients undergoing laparoscopic-assisted colon surgery [35], a result that was confirmed for open colorectal surgery by Nyström *et al*. [36]. Furthermore, several of these trials were underpowered. Taken together these conflicting results disallow a definitive answer whether wound-edge protectors constitute a reasonable approach to reduce SSI rates in general and abdominal surgery.

Hence, sufficient pilot data are available to justify the conduct of a prospective multicenter randomized patient-blinded and observer-blinded trial. Since further single center studies would not increase the external validity (generalizability) a multicenter approach was chosen and the trial was initiated within the Trial Network of the German Surgical Society (CHIR-*Net*). To further increase external validity broad inclusion criteria and few exclusion criteria are applied allowing for the screening and recruitment of many elective open general and abdominal surgical cases in the participating hospitals. Hospitals of different care levels participate in this trial together underlining the pragmatic approach of the trial. For many participating surgical departments SSIs represent the most frequent postoperative complication and thus a pressing surgical question that remains to be solved. To ensure data quality members of all participating centers are trained in GCP guidelines, trial intervention, documentation and blinding. In addition, internal validity is ensured by patient-blinding and observer-blinding, application of definite endpoints (SSI definition by the CDC) and complete outcome reporting and follow-up. Applying high methodological standards the results of the trial should help to improve surgical treatment of patients.

## Trial status

As of 29 February 2012 a total of 323 patients had been recruited to the trial.

## Competing interests

The authors declare that they have no competing interests.

## Authors’ contributions

ALM designed and planned the BaFO trial and wrote the manuscript. CWM designed and planned the BaFO and revised the manuscript. ME designed and planned the BaFO revised the manuscript. CRE designed and planned the BaFO revised the manuscript. CJ designed and planned the BaFO revised the manuscript. TS planned and wrote the statistical part of the BaFO trial. HF obtained funding, study design, revision of the manuscript. CS obtained funding, revision of the manuscript. JK obtained funding, study design, revision of the manuscript. All authors read and approved the final manuscript.

## References

[B1] Hawn MT Itani KM Gray SH Vick CC Henderson W Houston TK Association of timely administration of prophylactic antibiotics for major surgical procedures and surgical site infection J Am Coll Surg 2008 206 814 819 10.1016/j.jamcollsurg.2007.12.013 18471703

[B2] Belda FJ Aguilera L de la García-Asunción J Alberti J Vicente R Ferrándiz L Rodríguez R Company R Sessler DI Aguilar G Botello SG Ortí R Supplemental perioperative oxygen and the risk of surgical wound infection: a randomized controlled trial JAMA 2005 294 2035 2042 10.1001/jama.294.16.2035 16249417

[B3] Kurz A Thermal care in the perioperative period Best Pract Res Clin Anaesthesiol 2008 22 39 62 10.1016/j.bpa.2007.10.004 18494388

[B4] Kirkland KB Briggs JP Trivette SL Wilkinson WE Sexton DJ The impact of surgical-site infections in the 1990s: attributable mortality, excess length of hospitalization, and extra costs Infect Control Hosp Epidemiol 1999 20 725 730 10.1086/501572 10580621

[B5] Gastmeier P Brunkhorst F Schrappe M Kern W Geffers C How many nosocomial infections are avoidable? [in German] Dtsch Med Wochenschr 2010 135 91 93 10.1055/s-0029-1244823 20077383

[B6] Geffers C Rüden H Gesundheitberichterstattung des Bundes, Nosokomiale Infektionen [http://www.gbe-bund.de/gbe10/ergebnisse.prc_tab?fid=7845&suchstring=Heft_8&query_id=&sprache=D&fund_typ=TXT&methode=2&vt=1&verwandte=1&page_ret=0&seite=&p_lfd_nr=1&p_news=&p_sprachkz=D&p_uid=gast&p_aid=71600403&hlp_nr=3&p_janein=J]

[B7] Geffers C Sohr D Gastmeier P Mortality attributable to hospital-acquired infections among surgical patients Infect Control Hosp Epidemiol 2008 29 1167 1170 10.1086/592410 19014317

[B8] ECDC Annual epidemiological report on communicable diseases in Europe [http://ecdc.europe.eu/health_topics/HCAI]

[B9] Mangram AJ Horan TC Pearson ML Silver LC Jarvis WR Guideline for prevention of surgical site infection, Centres for Disease Control and Prevention (CDC) hospital infection control practices advisory committee Am J Infect Control 1999 1999 27 97 132 10196487

[B10] Page CP Bohnen JM Fletcher JR McManus AT Solomkin JS Wittmann DH Antimicrobial prophylaxis for surgical wounds. Guidelines for clinical care Arch Surg 1993 128 79 88 10.1001/archsurg.1993.01420130087014 8418785

[B11] Bratzler DW Houck PM Antimicrobial prophylaxis for surgery: an advisory statement from the National Surgical Infection Prevention Project Am J Surg 2005 189 395 404 10.1016/j.amjsurg.2005.01.015 15820449

[B12] Dellinger EP Gross PA Barrett TL Krause PJ Martone WJ McGowan JE Sweet RL Wenzel RP Quality standard for antimicrobial prophylaxis in surgical procedures. The Infectious Diseases Society of America Infect Control Hosp Epidemiol 1994 15 182 188 10.1086/646887 8207176

[B13] Napolitano LM Decolonization of the skin of the patient and surgeon Surg Infect 2006 7 Suppl 3 3 15

[B14] Itani KMF Wilson SE Awad SS Jensen EH Finn TS Abramson MA Ertapenem versus cefotetan prophylaxis in elective colorectal surgery N Engl J Med 2006 355 2640 2651 10.1056/NEJMoa054408 17182989

[B15] Milsom JW Smith DL Corman ML Howerton RA Yellin AE Luke DR Double-blind comparison of single-dose alatrofloxacin and cefotetan as prophylaxis of infection following elective colorectal surgery. Trovafloxacin Surgical Group Am J Surg 1998 176 Suppl 46 52 9935257 10.1016/s0002-9610(98)00220-7

[B16] Arnaud JP Bellissant E Boissel P Carlet J Chastang C Lafaix C Rio Y Berganeschi R Single-dose amoxycillin-clavulanic acid vs. cefotetan for prophylaxis in elective colorectal surgery: a multicentre, prospective, randomized study. The PRODIGE Group J Hosp Infect 1992 22 Suppl A 23 32 1362746 10.1016/s0195-6701(05)80004-0

[B17] Smith RL Bohl JK McElearney ST Friel CM Barclay MM Sawyer RG Foley EF Wound infection after elective colorectal resection Ann Surg 2004 239 599 605 10.1097/01.sla.0000124292.21605.99 15082963 PMC1356267

[B18] Darouiche RO Wall MJ Itani KMF Otterson MF Webb AL Carrick MM Miller HJ Awad SS Crosby CT Mosier MC Alsharif A Berger DH Chlorhexidine-alcohol versus povidone-iodine for surgical-site antisepsis N Engl J Med 2010 362 18 26 10.1056/NEJMoa0810988 20054046

[B19] Seiler CM Bruckner T Diener MK Papyan A Golcher H Seidlmayer C Franck A Kieser M Büchler MW Knaebel H-P Interrupted or continuous slowly absorbable sutures for closure of primary elective midline abdominal incisions: a multicentre randomized trial (INSECT: ISRCTN24023541) Ann Surg 2009 249 576 582 10.1097/SLA.0b013e31819ec6c8 19300233

[B20] Bennett-Guerrero E Pappas TN Koltun WA Fleshman JW Lin M Garg J Mark DB Marcet JE Remzi FH George VV Newland K Corey GR Gentamicin-collagen sponge for infection prophylaxis in colorectal surgery N Engl J Med 2010 363 1038 1049 10.1056/NEJMoa1000837 20825316

[B21] Auerbach AD Prevention of surgical site infections Making Health Care Safer: A Critical Analysis of Patient Safety Practices. Evidence Report/Technology Assessment 2001 Rockville, MD, USA: Agency for Healthcare Research and Quality 221 244

[B22] Coello R Glenister H Fereres J Bartlett C Leigh D Sedgwick J Cooke EM The cost of infection in surgical patients: a case–control study J Hosp Infect 1993 25 239 250 10.1016/0195-6701(93)90110-L 7907621

[B23] Kappstein I Schulgen G Fraedrich G Schlosser V Schumacher M Daschner FD Added hospital stay due to wound infections following cardiac surgery Thorac Cardiovasc Surg 1992 40 148 151 10.1055/s-2007-1020134 1412382

[B24] Poulsen KB Bremmelgaard A Sørensen AI Raahave D Petersen JV Estimated costs of postoperative wound infections. A case–control study of marginal hospital and social security costs Epidemiol Infect 1994 113 283 295 10.1017/S0950268800051712 7925666 PMC2271539

[B25] Merle V Germain JM Chamouni P Daubert H Froment L Michot F Teniere P Czernichow P Assessment of prolonged hospital stay attributable to surgical site infections using appropriateness evaluation protocol Am J Infect Control 2000 28 109 115 10.1016/S0196-6553(00)90018-X 10760218

[B26] Fry DE The economic costs of surgical site infection Surg Infect (Larchmt) 2002 3 Suppl 1 S37 S43 12573038 10.1089/sur.2002.3.s1-37

[B27] de Lissovoy G Fraeman K Hutchins V Murphy D Song D Vaughn BB Surgical site infection: incidence and impact on hospital utilization and treatment costs Am J Infect Control 2009 37 387 397 10.1016/j.ajic.2008.12.010 19398246

[B28] Broex ECJ van Asselt ADI Bruggeman CA van Tiel FH Surgical site infections: how high are the costs? J Hosp Infect 2009 72 193 201 10.1016/j.jhin.2009.03.020 19482375

[B29] KISS Surveillance System postoperative Wundinfektionen http://www.nrz-hygiene.de/surveillance/kiss/]

[B30] Reid K Pockney P Draganic B Smith SR Barrier wound protection decreases surgical site infection in open elective colorectal surgery: a randomized clinical trial Dis Colon Rectum 2010 53 1374 1380 10.1007/DCR.0b013e3181ed3f7e 20847618

[B31] Horiuchi T Tanishima H Tamagawa K Matsuura I Nakai H Shouno Y Tsubakihara H Inoue M Tabuse K Randomized, controlled investigation of the anti-infective properties of the Alexis retractor/protector of incision sites J Trauma 2007 62 212 215 10.1097/01.ta.0000196704.78785.ae 17215757

[B32] Sookhai S Redmond HP Deasy JM Impervious wound-edge protector to reduce postoperative wound infection: a randomised, controlled trial Lancet 1999 353 1585 10334259 10.1016/S0140-6736(99)00950-2

[B33] Kato Y Marusasa T Ichikawa S Lane GJ Okazaki T Yamataka A Lapprotector use decreases incisional wound infections in cases of perforated appendicitis: a prospective study Asian J Surg 2008 31 101 103 10.1016/S1015-9584(08)60068-8 18658006

[B34] Psaila JV Wheeler MH Crosby DL The role of plastic wound drapes in the prevention of wound infection following abdominal surgery Br J Surg 1977 64 729 732 10.1002/bjs.1800641012 922295

[B35] Kercher KW Nguyen TH Harold KL Poplin ME Matthews BD Sing RF Heniford BT Plastic wound protectors do not affect wound infection rates following laparoscopic-assisted colectomy Surg Endosc 2004 18 148 151 10.1007/s00464-003-8137-6 14625722

[B36] Nyström PO Broomé A Höjer H Ling L A controlled trial of a plastic wound ring drape to prevent contamination and infection in colorectal surgery Dis Colon Rectum 1984 27 451 453 10.1007/BF02555535 6378555

[B37] Anthony T Murray BW Sum-Ping JT Lenkovsky F Vornik VD Parker BJ McFarlin JE Hartless K Huerta S Evaluating an evidence-based bundle for preventing surgical site infection: a randomized trial Arch Surg 2011 146 263 269 10.1001/archsurg.2010.249 21079110

[B38] Fairclough JA Johnson D Mackie I The prevention of wound contamination by skin organisms by the pre-operative application of an iodophor impregnated plastic adhesive drape J Int Med Res 1986 14 105 109 3699240 10.1177/030006058601400210

[B39] Yoshimura Y Kubo S Hirohashi K Ogawa M Morimoto K Shirata K Kinoshita H Plastic iodophor drape during liver surgery operative use of the iodophor-impregnated adhesive drape to prevent wound infection during high risk surgery World J Surg 2003 27 685 688 10.1007/s00268-003-6957-0 12732986

[B40] Chiu KY Lau SK Fung B Ng KH Chow SP Plastic adhesive drapes and wound infection after hip fracture surgery Aust N Z J Surg 1993 63 798 801 10.1111/j.1445-2197.1993.tb00343.x 8274123

[B41] Dewan PA Van Rij AM Robinson RG Skeggs GB Fergus M The use of an iodophor-impregnated plastic incise drape in abdominal surgery-a controlled clinical trial Aust N Z J Surg 1987 57 859 863 10.1111/j.1445-2197.1987.tb01281.x 3326567

[B42] Webster J Alghamdi A Use of plastic adhesive drapes during surgery for preventing surgical site infection Cochrane Database Syst Rev 2007 4 CD006353 17943905 10.1002/14651858.CD006353.pub2

[B43] Pugh RN Murray-Lyon IM Dawson JL Pietroni MC Williams R Transection of the oesophagus for bleeding oesophageal varices Br J Surg 1973 60 646 649 10.1002/bjs.1800600817 4541913

[B44] International Conference on Harmonisation of Technical Requirements for Registration of Pharmaceuticals for Human Use ICH E6. Harmonised Guideline for Good Clinical Practice 1996 Geneva, Switzerland: ICH

[B45] Trotti A Colevas AD Setser A Rusch V Jaques D Budach V Langer C Murphy B Cumberlin R Coleman CN Rubin P CTCAE v3.0: development of a comprehensive grading system for the adverse effects of cancer treatment Semin Radiat Oncol 2003 13 176 181 10.1016/S1053-4296(03)00031-6 12903007

[B46] Culver DH Horan TC Gaynes RP Martone WJ Jarvis WR Emori TG Banerjee SN Edwards JR Tolson JS Henderson TS Surgical wound infection rates by wound class, operative procedure, and patient risk index. National Nosocomial Infections Surveillance System Am J Med 1991 91 152 157 10.1016/0002-9343(91)90361-Z 1656747

[B47] van Buuren S Multiple imputation of discrete and continuous data by fully conditional specification Stat Methods Med Res 2007 16 219 242 10.1177/0962280206074463 17621469

[B48] O’Brien PC Fleming TR A multiple testing procedure for clinical trials Biometrics 1979 35 549 556 10.2307/2530245 497341

[B49] International Conference on Harmonisation of Technical Requirements for Registration of Pharmaceuticals for Human Use ICH E9. Statistical Principles for Clinical Trials 1998 Geneva, Switzerland: ICH

[B50] World Medical Association Declaration of Helsinki - Ethical principles for medical research involving human subjects Proceedings of the 59th WMA General Assembly, Seoul, Korea, October 2008

[B51] Alexander JW Solomkin JS Edwards MJ Updated recommendations for control of surgical site infections Ann Surg 2011 253 1082 1093 10.1097/SLA.0b013e31821175f8 21587113

[B52] Melling AC Ali B Scott EM Leaper DJ Effects of preoperative warming on the incidence of wound infection after clean surgery: a randomised controlled trial Lancet 2001 358 876 880 10.1016/S0140-6736(01)06071-8 11567703

[B53] Kurz A Sessler DI Lenhardt R Perioperative normothermia to reduce the incidence of surgical-wound infection and shorten hospitalization. Study of Wound Infection and Temperature Group N Engl J Med 1996 334 1209 1215 10.1056/NEJM199605093341901 8606715

